# Kinetic Partitioning Modulates Human Telomere DNA G-Quadruplex Structural Polymorphism

**DOI:** 10.1371/journal.pone.0083420

**Published:** 2013-12-18

**Authors:** Xi Long, Michael D. Stone

**Affiliations:** 1 Department of Chemistry and Biochemistry, University of California Santa Cruz, Santa Cruz, California, United States of America; 2 Center for Molecular Biology of RNA, University of California Santa Cruz, Santa Cruz, California, United States of America; University of Oklahoma, United States of America

## Abstract

Telomeres are specialized chromatin structures found at the end of chromosomes and are crucial to the maintenance of eukaryotic genome stability. Human telomere DNA is comprised of the repeating sequence (T_2_AG_3_)_n_, which is predominantly double-stranded but terminates with a 3’ single-stranded tail. The guanine-rich tail can fold into secondary structures known as a G-quadruplexes (GQs) that may exist as a polymorphic mixture of anti-parallel, parallel, and several hybrid topological isomers. Using single-molecule Förster resonance energy transfer (smFRET), we have reconstructed distributions of telomere DNA GQ conformations generated by an *in situ* refolding protocol commonly employed in single-molecule studies of GQ structure, or using a slow cooling DNA annealing protocol typically used in the preparation of GQ samples for ensemble biophysical analyses. We find the choice of GQ folding protocol has a marked impact on the observed distributions of DNA conformations under otherwise identical buffer conditions. A detailed analysis of the kinetics of GQ folding over timescales ranging from minutes to hours revealed the distribution of GQ structures generated by *in situ* refolding gradually equilibrates to resemble the distribution generated by the slow cooling DNA annealing protocol. Interestingly, conditions of low ionic strength, which promote transient GQ unfolding, permit the fraction of folded DNA molecules to partition into a distribution that more closely approximates the thermodynamic folding equilibrium. Our results are consistent with a model in which kinetic partitioning occurs during *in situ* folding at room temperature in the presence of K^+^ ions, producing a long-lived non-equilibrium distribution of GQ structures in which the parallel conformation predominates on the timescale of minutes. These results suggest that telomere DNA GQ folding kinetics, and not just thermodynamic stability, likely contributes to the physiological ensemble GQ structures.

## Introduction

**Figure 1:**
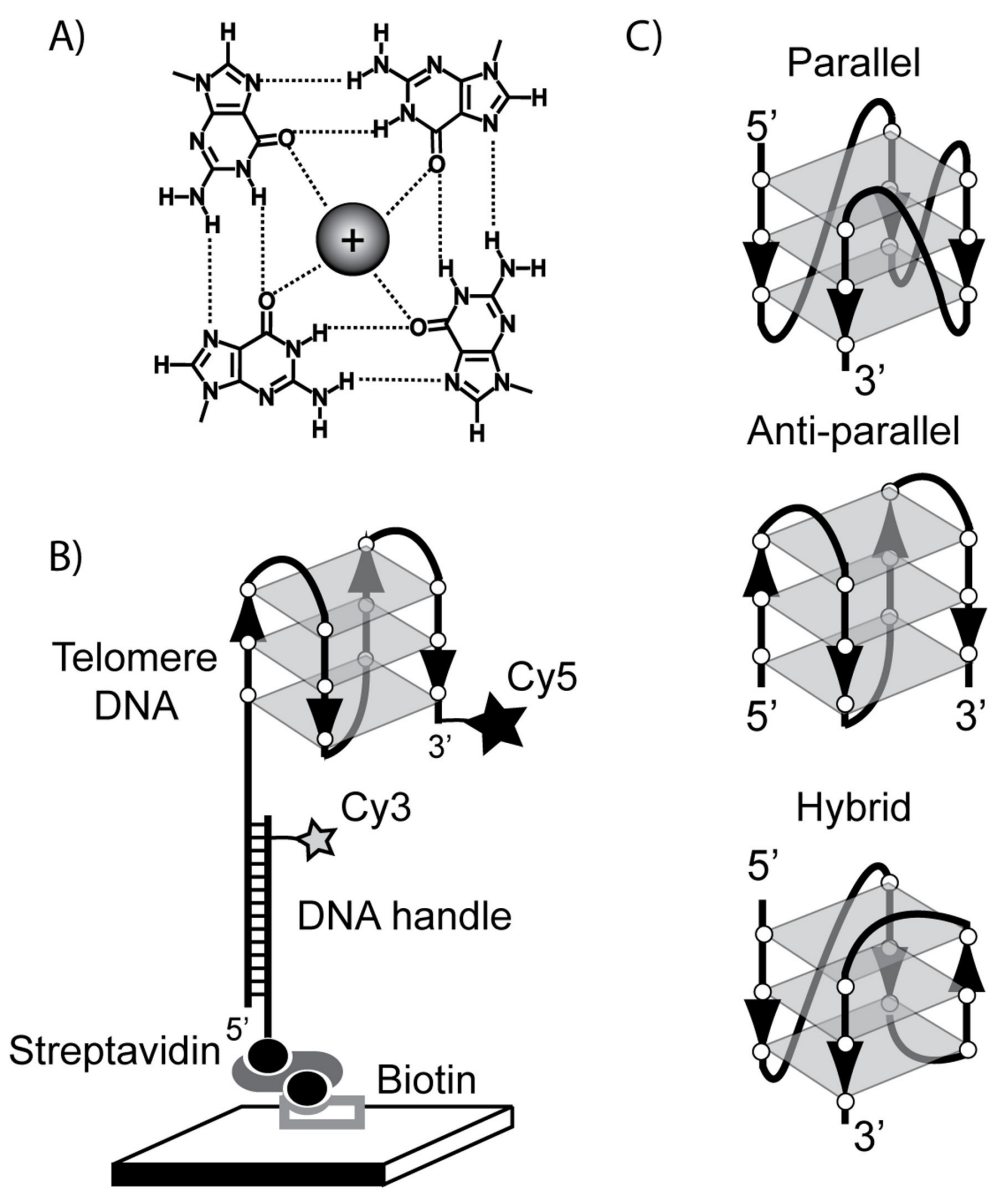
Experimental setup for telomere DNA GQ single molecule FRET measurements. (A) Planar telomere DNA G-quartet structure with coordinated monovalent cation. (B) Grey rectangles represent planar G-quartets and backbone polarity (5’à3’) is indicated by black arrowheads. Molecules are immobilized on a microscope slide and FRET is measured as the energy transfer between the donor (Cy3) and acceptor (Cy5) dye. (C) Human telomere G-quadruplex structures formed by Tel23 sequence.

The first solution structure of a human telomere G-quadruplex (GQ) revealed a fundamental structural architecture in which guanine bases are hydrogen bonded in a planar quartet geometry (Figure 1A) [1].Three consecutive intra-molecular G-quartets may interact via stacking interactions and are topologically linked by short intervening DNA loop sequences (Figure 1B). In addition, telomere DNA GQ structures are stabilized by monovalent cations that may be site-specifically coordinated at the center of the planar G-quartet motif [1]. The specific identity of the monovalent cation used during telomere DNA GQ folding has a pronounced impact on the distribution of observed structures in a variety of model systems [2-8]. In the case of human telomere DNA, Na^+^ ions promote the formation of the anti-parallel basket type GQ conformation, while K^+^ ions induce folding of a mixture of GQ structures, including the parallel and anti-parallel conformations, as well as several hybrid topological forms [9,10] (Figure 1C). Since K^+^ is the predominant monovalent cation *in vivo*, much interest has focused on understanding telomere DNA GQ structural polymorphism induced by K^+^ ions. 

Human telomere DNA GQ structure has been studied by numerous techniques including NMR [1,11-14], X-ray crystallography [15], circular dichroism [16-19], UV melting [13,20,21], force spectroscopy [22-24], and Förster Resonance Energy Transfer (FRET) [25-28]. These studies revealed telomere DNA GQs are highly polymorphic, assuming a variety of topologically distinct structural forms, which may interconvert under certain experimental conditions. The dynamic properties of telomere DNA GQs motivated the use of single molecule FRET (smFRET) to provide a detailed description of GQ structural heterogeneity and folding [27-30]. However, many single molecule analyses of telomere DNA GQ structure employed sample preparation protocols that differ in potentially substantial ways from protocols utilized in prior ensemble biophysical studies. Therefore, in the present study we have used smFRET to systematically investigate the impact of different DNA folding protocols on the observed distribution of telomere DNA conformations. Our results demonstrate that the distribution of telomere DNA conformations measured by smFRET is dramatically altered by changes in the DNA folding protocol under otherwise identical buffer conditions. By analyzing the kinetics of GQ DNA folding at room temperature over long timescales (from minutes to hours), as well as in varying ionic strengths, we provide evidence that kinetic partitioning produces a long-lived non-equilibrium distribution of GQ structures. These results not only highlight the importance of the specific folding protocol being used during biophysical analyses of GQ structure, but also suggest that kinetics of folding may dictate the distribution of telomere DNA GQs present *in vivo*. 

## Materials and Methods

### Dye Labeling of DNA oligonucleotides

All DNA fragments were purchased from Integrated DNA Technologies, Inc. (Table 1).

**Table 1:** DNA oligonucleotides used in this study.

Tel22	5’GCGTGGCACCGGTAATAGGAAAATGGAGAAGGGTTAGGGTTAGGGTTAGGG (Amino C3) -3’
Tel23	5’GCGTGGCACCGGTAATAGGAAAATGGAGATAGGGTTAGGGTTAGGGTTAGGG (Amino C3) -3’
Hybrid mutant	5’GCGTGGCACCGGTAATAGGAAAATGGAGATTGGGTTAGGGTTAGGGTTAGGGA (Amino C3)-3’
DNA handle	5’ TCTCCAT(Amino C6 T)TTCCTATTACCGGTGCCACGC-Biotin 3’
Tel23 no duplex	5’ TAGGGTTAGGGTTAGGGTTAGGG 3’
Hybrid mutant no duplex	5’ TTGGGTTAGGGTTAGGGTTAGGGA 3’

The 3’amino modified, Tel22, Tel23, and Hybrid mutant oligonucleotides were labeled with mono-reactive Cy5 (GE Healthcare) and the internally amino modified biotinylated DNA handle was labeled with mono-reactive Cy3 (GE Healthcare). Dye labeled fragments were, EtOH precipitated and purified by reverse-phase chromatography on a C8 column (Agilent, Eclipse XDB-C8) on an AKTA purifier. Following HPLC purification samples were EtOH precipitated, and resuspended in ddH_2_O. DNA concentrations were determined using a Nanodrop. 

### DNA annealing reactions

Cy5 labeled Tel22, Tel23 and Hybrid Mutant were annealed to Cy3 labeled biotinylated DNA handle by heating to 95 °C for 4 minutes followed by slow cooling to room temperature (over several hours) in the presence of a buffer (10 mM Tris-HCl pH 8) containing either 100 mM KCl or 100 mM NaCl (as described in the paper).

### Single molecule FRET measurements

Quartz slides (Finkenbeiner Inc.) were cleaned by sonicating for 20 minutes in 10% w/v Alconox, 20 minutes in ddH_2_O, 20 minutes in acetone, 20 minutes 1M KOH, then 20 minutes in fresh 1M KOH. Slides are then rinsed with ddH2O and dried under nitrogen, followed by flame cleaning with a propane torch for ~2 minutes. Sample chambers were prepared by sandwiching pieces of parafilm between the quartz slide and a plasma cleaned coverglass (Harrik Plasma Cleaner), cover glasses from TED Pella, Inc. (Prod.# 260146). Chambers were heated on a hot block (95°C) for 1 minute to seal the parafilm to the glass. Channels (~10 uL total volume) were treated with 35 uL of 1 mg/mL of biotinylated BSA (Sigma Cat.#A8549) for 5 minutes, washed with 100 uL of T50 buffer (10 mM Tris-HCl pH 8, 50 mM NaCl), then incubated with 50uL of 0.2 mg/mL streptavidin (Invitrogen Cat.#S888), then washed with 100 uL of T50. Channels were then equilibrated with buffer matching the desired experimental condition (see main text). Next, 100 uL of 5-10 pM annealed biotinylated - DNA were deposited onto the streptavidin coated quartz slide in the desired buffer condition. After 5 minutes incubation, 100 uL of a buffer containing either 100 mM KCl or 100 mM NaCl was flushed to the slide to remove the unbound DNA. Data was collected in imaging buffer containing 10mM Tris pH 8, 100 mM KCl or NaCl, 0.4% (w/v) D-glucose, 0.1 mg/ml glucose oxidase (Sigma Cat.# G2133-250KU), 0.02 mg/ml catalase (CalBiochem Cat.#219001) and saturated with Trolox (Aldrich Cat.#23,881-3). Data was acquired using a green laser (532 nm, Laserglow, Inc.) and prism-type total internal reflection microscopy on an inverted Olympus IX71 microscope equipped with an Andor IXON (897) CCD camera with 100 ms integration time. For *in situ* refolding experiments, surface immobilized molecules incubated for 5 minutes in buffer containing no KCl or NaCl (10 mM Tris pH 8), followed by an incubation in the presence of 100 mM KCl for the indicated time. For experiments analyzing the *in situ* refolding distributions under varying KCl concentrations, folding incubation times were fixed at 10 minutes, and smFRET measurements were performed at the indicated salt concentrations in the presence of 50 mM Tris pH 8. smFRET histograms were recorded as described below.

### smFRET analysis

Raw movie files were analyzed using in-house written software available upon request (IDL and Matlab). FRET is defined as the efficiency of energy transfer between acceptor and donor dye, I_A_/(I_A_+I_D_). I_A_ is the acceptor intensity and I_D_ is the donor intensity. FRET histograms were compiled from the average FRET value obtained from each molecule over a 2 second observation time. The total data collection time for each smFRET histogram is approximately one minute. In each experiment a FRET = 0 population is observed, derived from molecules harboring only an active FRET donor dye. The origin of the FRET = 0 peak was confirmed by direct excitation of the acceptor molecules with a red laser (657 nm, Vortran Inc.). For clarity, these zero FRET peaks have been removed from the figures shown in the paper. To fit the smFRET distributions, data were binned (bin size was FRET = 0.03) and fit with multiple Gaussian functions using Origin. Based upon previous characterization of our system, the distribution widths were fixed at a value of 0.08 to avoid fitting artifacts. Fitting was evaluated using the adjusted R^2^ value to determine the best fit model. 

### CD measurements

The DNA fragments (Tel 23 no duplex, and Hybrid mutant no duplex) used in the experiment were ordered from Integrated DNA Technologies,Inc. The CD spectra were measured by using an AVIV Model 62DS Circular Dichroism Spectrometer. The DNA sample was diluted in a buffer (10 mM Tris pH8) containing either 100 mM KCl or 100 mM NaCl, heated to 95 °C for 4 minutes followed by slow cooling to room temperature. 300 uL of 300 uM DNA solution was contained in a quartz cell of 1 mm optical path length and instrument scanning speed of 1mm per minute with 8 seconds response time. The spectra were averages of three repetitive scans between 200 and 340 nm at room temperature with buffer scan baseline subtraction and smoothed with a Savitsky-Golay filter [31].

## Results

### Distinct DNA folding protocols alter the observed distribution of GQ structures

To analyze telomere DNA GQ folding, we designed a smFRET construct comprised of the model telomere DNA sequence TAGGG(T_2_AG_3_)_3_. This 23 nucleotide telomere DNA substrate (Tel23) was shown previously to adopt a variety of GQ topologies, including the parallel, anti-parallel, and several hybrid conformations [11,12] (Figure 1C). To mimic the natural double-stranded/single-stranded telomere DNA junction, we designed the construct to have a single-stranded DNA G-rich tail that runs in the 5’à3’ direction with respect to the phosphodiester backbone. We introduced a donor FRET dye (Cy3) on a biotinylated DNA handle, which is complementary to a 5’ non-telomeric extension of the G-rich telomere DNA strand. In addition, we modified the 3’ terminus of the G-rich strand with an acceptor FRET dye (Cy5) (Figure 1B). Single-molecule FRET labeled Tel23 molecules were surface immobilized via a biotin-streptavidin linkage and imaged using prism-type total internal reflection microscopy [32]. In our smFRET assay the conformational properties of telomere DNA are monitored as the efficiency of energy transfer between the donor and acceptor dye, and we define FRET = *I*_*A*_/(*I*_*A*_+ *I*_*D*_ **), where *I*_*A*_ and *I*_*D*_ are the background corrected intensities of the acceptor and donor dyes, respectively. 

**Figure 2:**
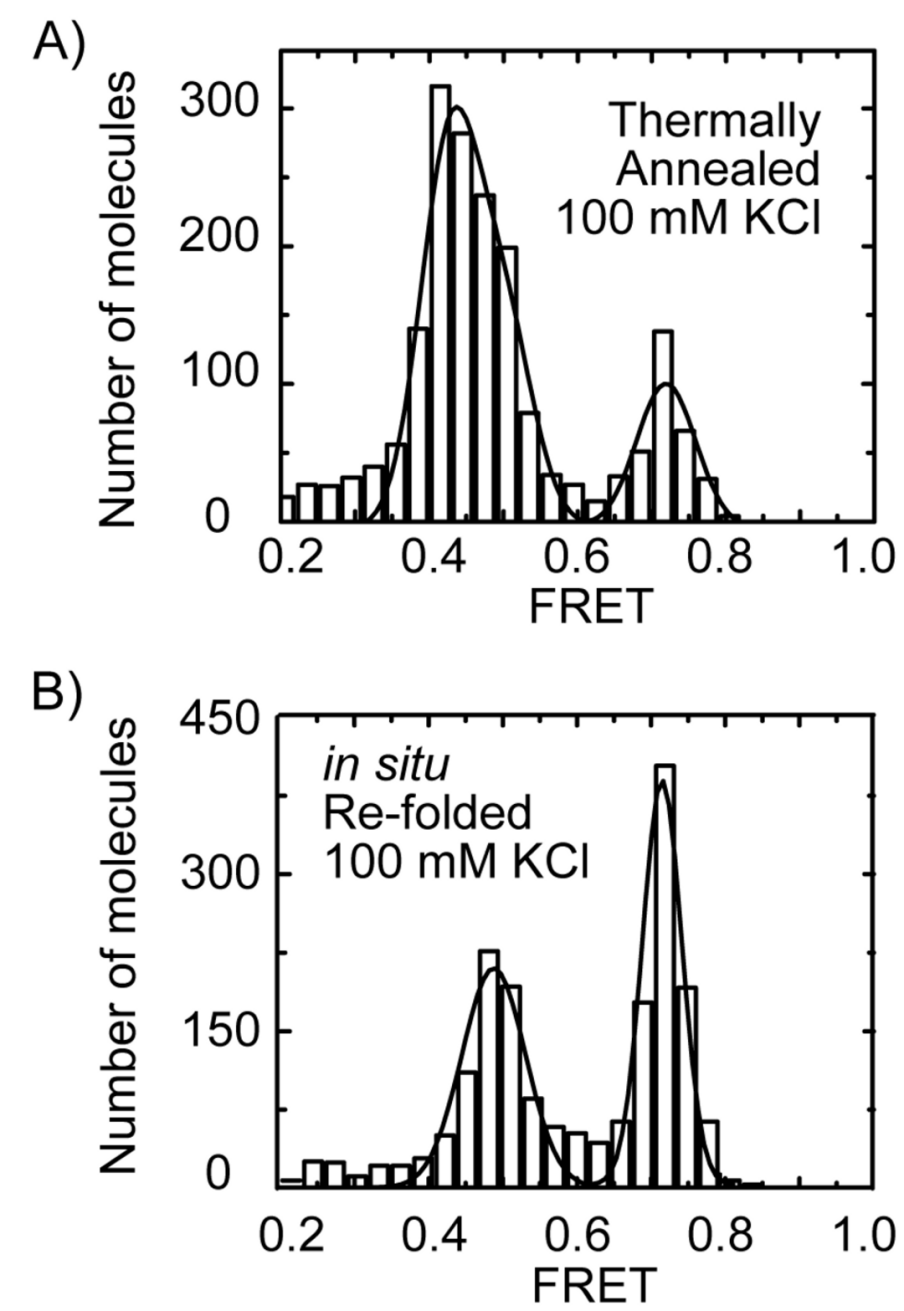
Single-molecule FRET histograms for telomere DNA GQ constructs. Gaussian fits to the data are shown in black. (A)Tel23 thermally annealed in 100 mM KCl. (B) Tel23 *in* *situ* refolded in 100 mM KCl.

Our initial characterization of the Tel23 smFRET construct employed a DNA folding protocol used in previously reported NMR and CD spectroscopic analyses [12]. In this approach (hereafter referred to as *thermal annealing*), Tel23 molecules were heated to 95°C for 4 minutes in the presence of 100 mM KCl and equimolar amounts of biotinylated DNA handle, then allowed to slowly equilibrate to room temperature over a period of several hours. These conditions are sufficient to melt out any pre-existing GQ structures [33] and promote the base paring of the biotin-labeled DNA handle. Following thermal annealing, Tel23 molecules were surface immobilized and imaged in buffer containing 100 mM KCl. The smFRET distributions constructed from Tel23 molecules thermally annealed and imaged in the presence of 100 mM KCl possessed a broad mid-FRET population centered at FRET = 0.44 and a second higher FRET population centered at FRET = 0.72 (Figure 2A). Moreover, thermal annealing of Tel23 molecules in the presence of 20 mM KCl yielded a similar FRET distribution (Figure S1 in File S1), demonstrating the final equilibrium of folded GQ structures is independent of salt concentration.

Having characterized the structural distributions generated by thermal annealing of Tel23 molecules in KCl, we next studied Tel23 *in situ* refolding in KCl. Surface immobilized Tel23 molecules initially folded by thermal annealing in 100 mM KCl were incubated in the absence of salt to promote DNA unfolding, producing a single unfolded FRET population centered at FRET = 0.23 (Figure S2 in File S1) as previously described [29]. We then attempted to refold the telomere DNA molecules *in situ* by introducing buffer containing 100 mM KCl at room temperature and incubating for 10 minutes. Interestingly, the smFRET histogram generated by *in situ* KCl folding was markedly altered from the KCl thermal annealing experiments, exhibiting a predominant high FRET population centered at FRET = 0.72 and second population centered at FRET = 0.50 (compare Figure 2A with 2B). Taken together, these results demonstrate the distribution of structures generated by KCl thermal annealing of Tel23 is significantly altered during *in situ* KCl refolding at room temperature on the time scale of minutes. 

### *In situ* refolding induces kinetic trapping of GQ conformations

**Figure 3:**
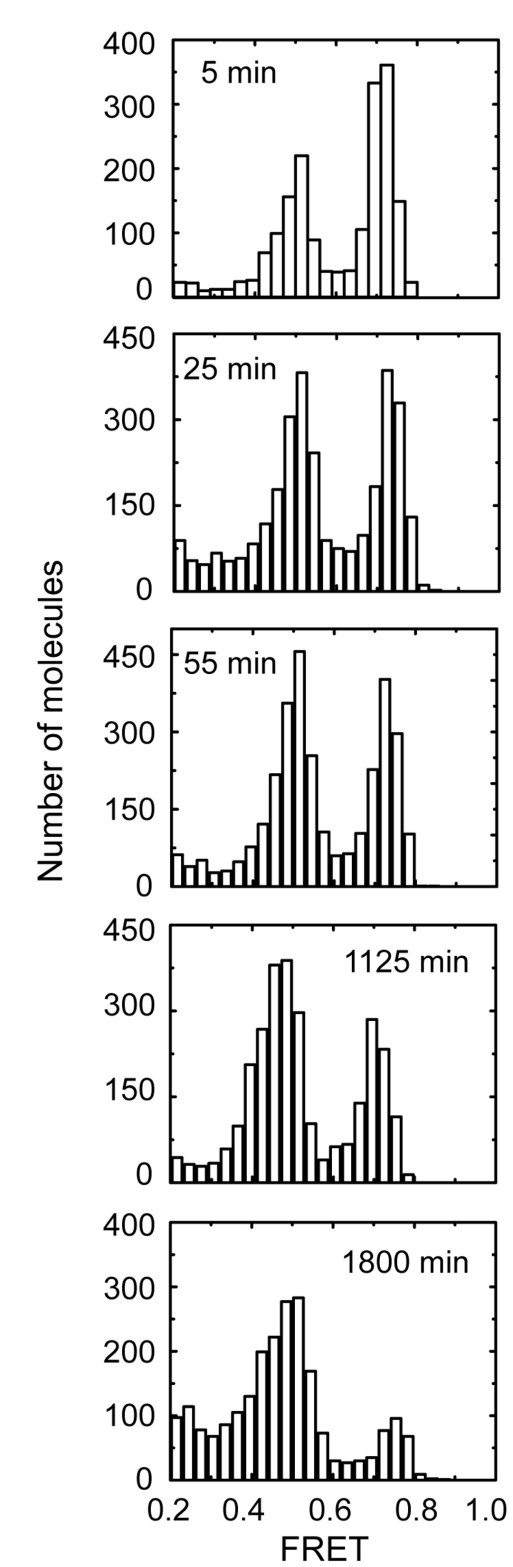
Single-molecule FRET histograms of Tel23 *in situ* refolded in 100 mM KCl for the indicated period of time.

One model to explain the differences observed for Tel23 molecules folded by either KCl *in situ* refolding or thermal annealing is that during *in situ* refolding the DNA becomes kinetically trapped in a higher energy GQ conformation, and if given sufficient time to equilibrate, the system would ultimately reach thermodynamic equilibrium. To test this notion, we performed a time course on KCl *in situ* refolded molecules from minutes to hours (Figure 3). Indeed, we found that over the course of several hours, the smFRET distributions of Tel23 molecules in situ refolded in 100 mM KCl gradually converted to a distribution that more closely resembled the KCl thermal annealing result, characterized by a depletion of the high FRET peak and a corresponding increase in abundance of the mid FRET state (compare Figure 2A and Figure 3, bottom panel). Moreover, time course experiments performed at two lower KCl concentrations (20 mM and 60 mM), revealed the convergence of the smFRET distributions to the same states, albeit with slightly different kinetics (Figure S3 in File S1). These results suggest that the KCl *in situ* refolding protocol kinetically traps the high FRET conformation which can, if given sufficient time, partition into the more energetically stable mid-FRET conformation. 

### Ionic strength alters the GQ folding landscape

**Figure 4:**
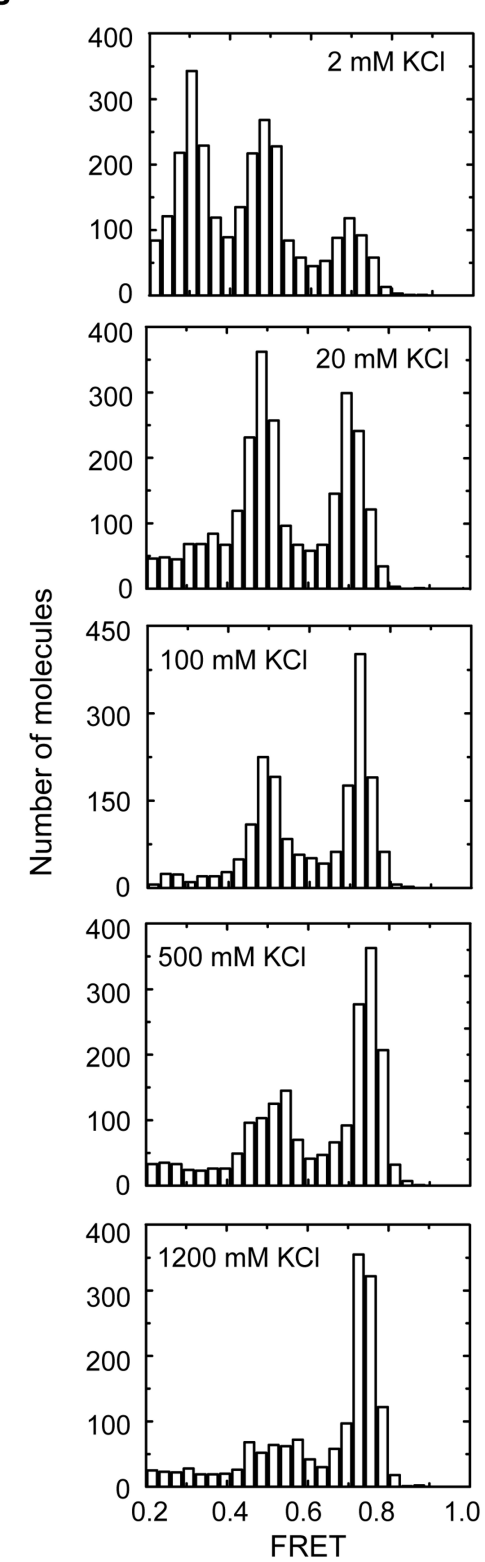
Single-molecule FRET histograms of Tel23 *in* *situ* refolded at the indicated KCl concentrations.

We next analyzed the impact of KCl concentration on the observed smFRET distributions after 10 minutes of *in situ* refolding. As described above, previous reports demonstrated that conditions of low ionic strength promote GQ unfolding resulting in a low FRET population (Figure S2 in File S1) [29]. Interestingly, we find that at low KCl concentration, where GQ unfolding is favored, the distribution of folded states more closely resembles the distribution obtained by thermal annealing at 100 mM KCl (Figure 4, top panel). In contrast, elevated KCl concentrations produce distributions of GQ structures with a larger fraction in the kinetically trapped (high-FRET) conformation (Figure 4, bottom panels). These experiments are consistent with previous studies of telomere DNA structural dynamics at low KCl concentrations, in which the unfolded conformation (low FRET state) was shown to be an obligatory intermediate during interconversion of distinct GQ topological isomers [27]. Therefore, the ability of the DNA to sample an unfolded conformation at low ionic strength increases the rate at which the folded fraction can attain the thermodynamically favored structural equilibrium. 

### The parallel GQ conformation is the kinetically trapped structure

**Figure 5:**
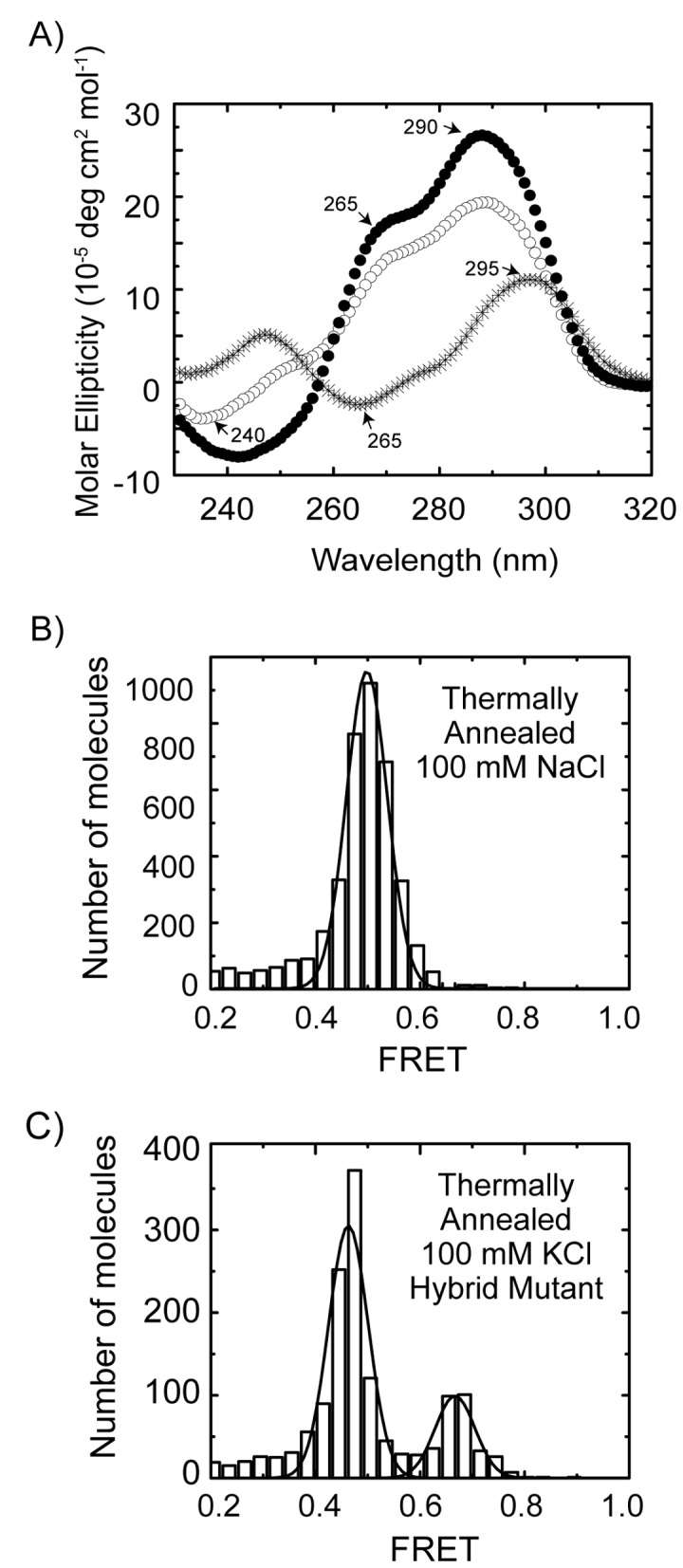
Structural assignments of distinct FRET states. (A) CD spectra of Tel23 thermally annealed in 100 mM KCl (open circles), Tel23 in 100 mM NaCl (asterisks), and Hybrid Mutant in 100 mM KCl (closed circles). (B) The smFRET distribution of Tel23 thermally annealed in 100 mM NaCl fit with Gaussian functions. Gaussian fits to the data are shown in black. (C) The smFRET distribution of Hybrid Mutant thermally annealed in 100 mM KCl fit with Gaussian functions. Gaussian fits to the data are shown in black.

We note that smFRET measurements alone do not provide sufficient resolution to unambiguously resolve all of the distinct GQ conformations anticipated to coexist for Tel23 folded in KCl. However, circular dichroism (CD) measurements on the telomere DNA constructs used in our smFRET measurements folded in the presence of either KCl or NaCl permit us to make preliminary correlations between particular GQ conformations and the FRET states observed in our experiments. A CD spectrum taken on Tel23 molecules prepared by KCl thermal annealing exhibited a positive peak at ~290 nm, a shoulder at ~265 nm, and a small negative peak at ~240 nm (Figure 5A, open circles). These CD signatures indicate a mixture of telomere DNA GQ topologies including anti-parallel, hybrid and parallel conformations, consistent with NMR studies of Tel23 molecules [34,35]. Our smFRET measurements of Tel23 prepared by KCl thermal annealing revealed the presence of at least two distinct FRET states. Interestingly, treating the broad mid-FRET peak as two distinct populations, FRET = 0.42 and FRET = 0.50, yielded a substantially better fit as judged by adjusted R^2^ values (Figure S4 in File S1), suggesting the mid-FRET peak may represent a superimposition of two distinct FRET states. In contrast, smFRET analysis of a shorter telomere DNA substrate lacking the 5’ terminal thymine, Tel22 (AG_3_(T_2_AG_3_)_3_), prepared by KCl thermal annealing yielded one predominant mid-FRET state (Figure S4 in File S1). This observation is consistent with a previously reported NMR structure, which demonstrated a requirement for a Watson-Crick base pair between the 5’ terminal thymine and an internal DNA loop to stabilize an alternative hybrid GQ conformation [12]. 

We next analyzed the conformational properties of Tel23 in the presence of 100 mM NaCl, a condition that promotes homogeneous folding of telomere DNA into the anti-parallel GQ conformation [1]. As anticipated, the CD spectrum of Tel23 thermally annealed in the presence of 100 mM NaCl yielded a strong positive peak at ~295 nm and a negative peak at ~265 nm, indicative of the anti-parallel conformation (Figure 5A, asterisks) [36]. When analyzed by smFRET, Tel23 molecules thermally annealed and imaged in 100 mM NaCl produced a distribution exhibiting a single predominant population centered on FRET=0.50 (Figure 5B). Based upon these results we conclude the single major population centered at FRET = 0.50 in our smFRET experiments corresponds to the anti-parallel GQ conformation. 

To further dissect the heterogeneity of the smFRET distributions observed for Tel23 prepared by KCl thermal annealing, we next utilized a mutant telomere DNA construct that promotes the hybrid GQ conformation [12]. The CD spectrum for the hybrid mutant prepared by KCl thermal annealing was similar to Tel23 treated in the same manner, but possessed a more prominent positive peak at ~ 290 nm, a pronounced shoulder at ~ 265 nm, and a strong negative peak at ~240 nm (Figure 5A, closed circles). When analyzed by smFRET, the hybrid mutant folded under KCl thermal annealing conditions yielded a smFRET distribution possessing a predominant population in the mid-FRET range (Figure 5C). Thus, although Tel23 molecules folded by NaCl thermal annealing and the hybrid mutant prepared by KCl thermal annealing yield FRET distributions with overlapping mid-FRET populations (compare Figure 5B and 5C), their respective CD spectra clearly indicate a difference in the predominant GQ conformation (Figure 5A). Thus, we suggest the hybrid and anti-parallel GQ conformations are both characterized by a mid-FRET state, while the high FRET kinetically trapped state we observe in our experiments represents the parallel conformation. In further support of these assignments, a qualitative analysis of the atomic resolution structures for each of these telomere GQ conformations indicates that the DNA termini are in much closer proximity in the parallel conformation when compared with both the anti-parallel and hybrid structures (Figure S5 in File S1).

## Discussion

**Figure 6:**
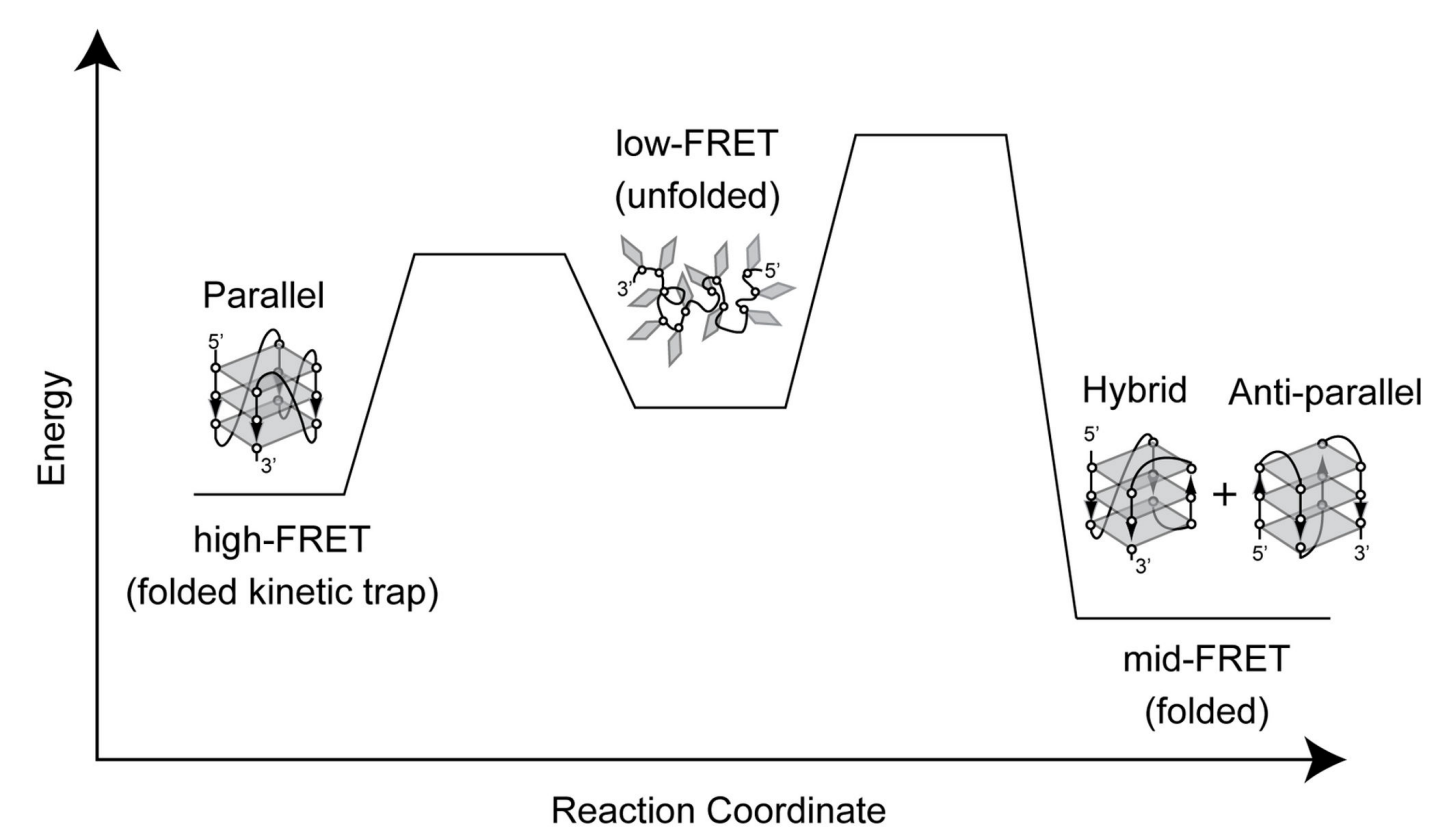
A qualitative energy landscape model for kinetic partitioning during telomere DNA GQ folding. A lower energy barrier between the low-FRET (unfolded) and the high-FRET (folded) state creates a kinetic trap during the early stages of folding. To escape the kinetic trap, the molecule must unfold and then re-fold into one of the more energetically stable mid-FRET (folded) states. This process is facilitated during thermal annealing at higher temperatures, or during *in* *situ* folding by low ionic strength or prolonged incubation times.

In summary, we have used smFRET to directly demonstrate the distribution of telomere DNA GQ structures generated during KCl *in situ* refolding on the timescale of minutes is demonstrably altered from the identical molecules prepared by KCl thermal annealing. Since thermally annealed and *in situ* refolded Tel23 molecules were imaged under identical buffer conditions (100 mM KCl), we conclude that *in situ* refolding gives rise to kinetic partitioning of GQ conformations. A combination of smFRET and CD measurements on several model telomere DNA constructs in the presence of KCl or NaCl provided insight into which GQ conformations correspond to the observed FRET states. Since we cannot conclusively differentiate between the two overlapping mid-FRET conformations, which we suggest represent a mixture of the hybrid and anti-parallel GQ folds, we interpret our results in terms of a simple three state model: low-FRET (unfolded), mid-FRET (anti-parallel and/or hybrid GQ), and high-FRET (parallel GQ) (Figure 6). Kinetic partitioning of GQ folds can be understood by assuming there is a large energy barrier between each of the folded and unfolded GQ states. Thus, high temperature or low ionic strength conditions serve to reduce the height of the barriers separating these states, and in turn facilitate a more rapid attainment of the thermodynamically favored GQ conformations. Additional experiments will be required to investigate the contribution of kinetic partitioning during telomere DNA GQ folding *in vivo*, as well as the potential impact of GQ resolving enzymes on the observed distributions of GQ conformations.

## Supporting Information

File S1 Supporting figures. Figure S1, Gaussian fitting of smFRET distribution of Tel23 molecules thermally annealed and imaged in 20 mM KCl. Figure S2, *in* *situ* unfolding of thermally annealed Tel23 molecules. Figure S3, Single-molecule FRET histograms of Tel23 *in* *situ* refolded in 60mM and 20mM KCl for the indicated period of time. Figure S4, Gaussian fitting of Tel23 and Tel22 smFRET distributions. Figure S5, Atomic resolution structures of the parallel, anti-parallel, and a hybrid telomere DNA GQ conformations.
(DOCX)
