# Supplementary material for: Kinetic Partitioning Modulates Human Telomere DNA G-Quadruplex Structural Polymorphism
Source: PLoS One. 2013 Dec 18;8(12):e83420. doi: 10.1371/journal.pone.0083420 (PMC3867459; doi:10.1371/journal.pone.0083420)
Supplement: File S1 — Supporting figures. Figure S1, Gaussian fitting of smFRET distribution of Tel23 molecules thermally annealed and imaged in 20 mM KCl. Figure S2, in situ unfolding of thermally annealed Tel23 molecules. Figure S3, Single-molecule FRET histograms of Tel23 in situ refolded in 60mM and 20mM KCl for the indicated period of time. Figure S4, Gaussian fitting of Tel23 and Tel22 smFRET distributions. Figure S5, Atomic resolution structures of the parallel, anti-parallel, and a hybrid telomere DNA GQ conformations. (DOCX) [file pone.0083420.s001.docx]

**Supporting Information for**

**Kinetic partitioning modulates human telomere DNA G-quadruplex structural polymorphism**

**Xi Long^+^ and Michael D. Stone^+,§^***

^+^Department of Chemistry and Biochemistry, University of California Santa Cruz, Santa Cruz, California, USA

^§^Center for Molecular Biology of RNA, University of California Santa Cruz, Santa Cruz, California, USA

*corresponding author; email: [mds@ucsc.edu](mailto:mstone@chemsitry.ucsc.edu)

**Figure S1**


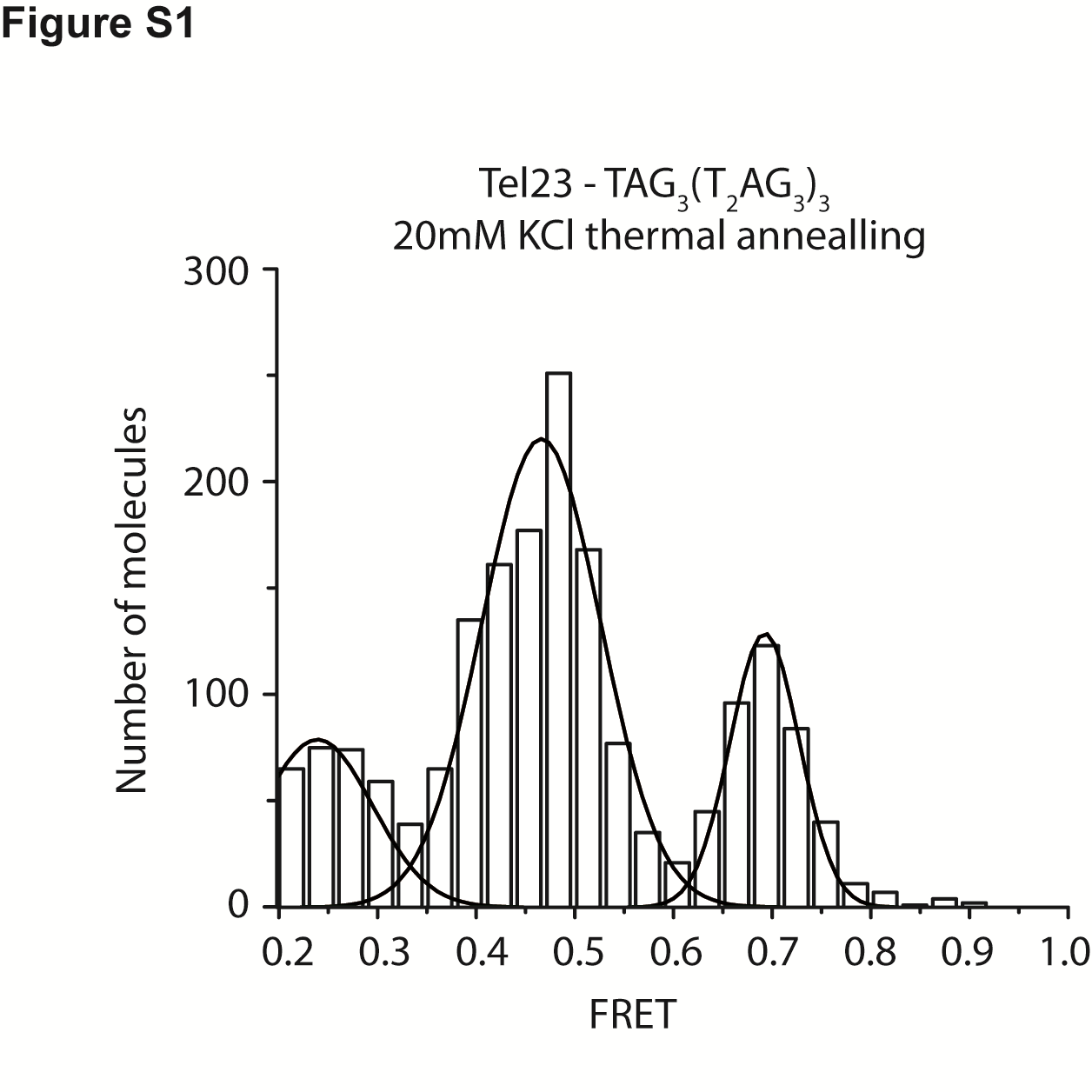


**Figure S1.** Gaussian fitting of smFRET distribution of Tel23 molecules thermally annealed and imaged in 20 mM KCl fit with three Gaussian functions centered at FRET = 0.25 (unfolded population), FRET=0.49 and FRET = 0.72 . The fit was performed using nonlinear least-squares curve fitting in Origin. Indicated R^2^ values are adjusted for the number of free parameters used in the fit.

**Figure S2**


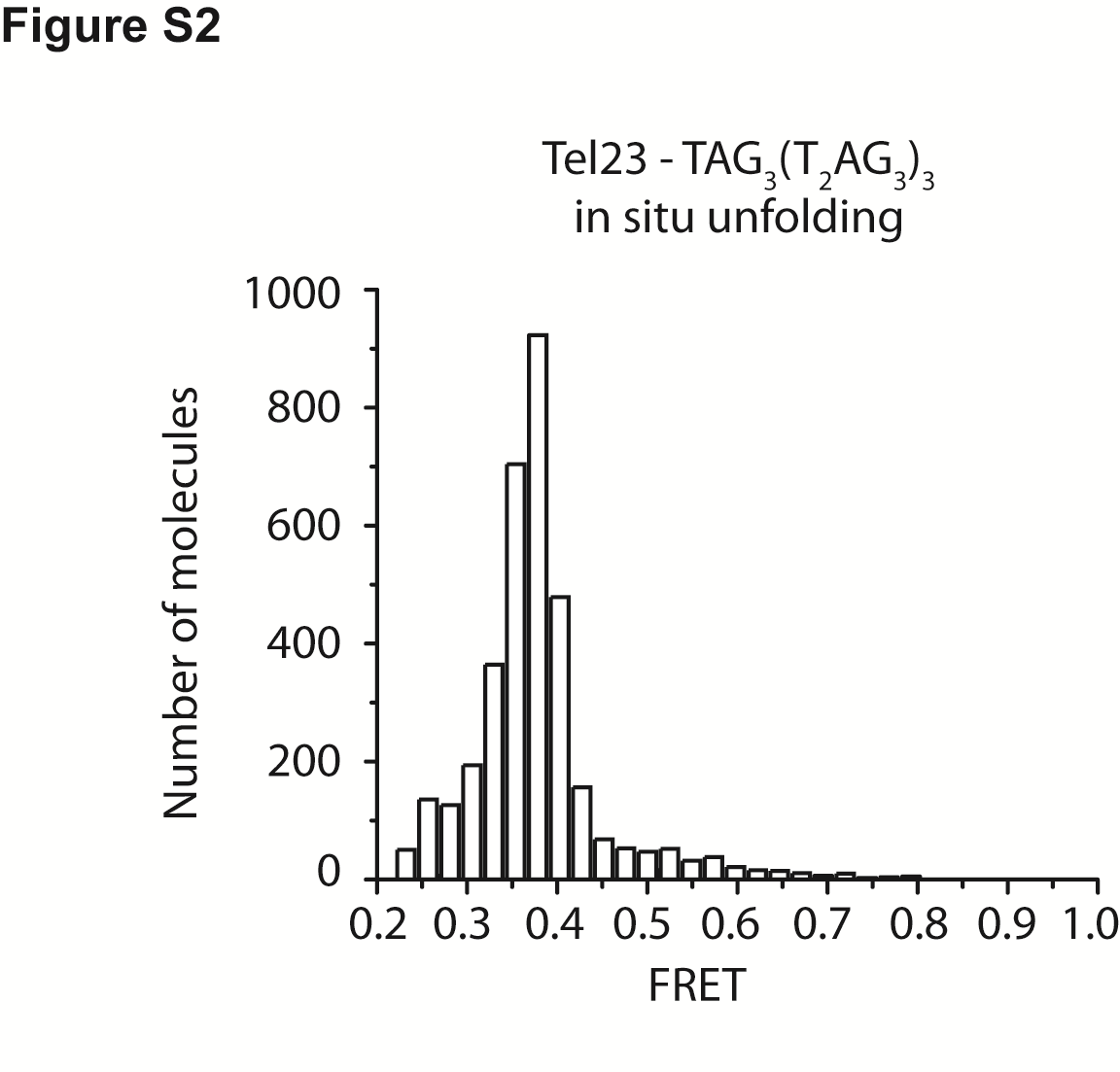


**Figure S2.** *In situ* unfolding of thermally annealed Tel23 molecules. Surface immobilized Tel 23 molecules that were thermally annealed and imaged in buffer T0 (10 mM Tris pH8, no salt). The unfolding treatment resulted in smFRET distributions that were centered at a value of FRET = 0.23, indicative of the unfolded state as described previously[[1](#_ENREF_2)].

**Figure S3**


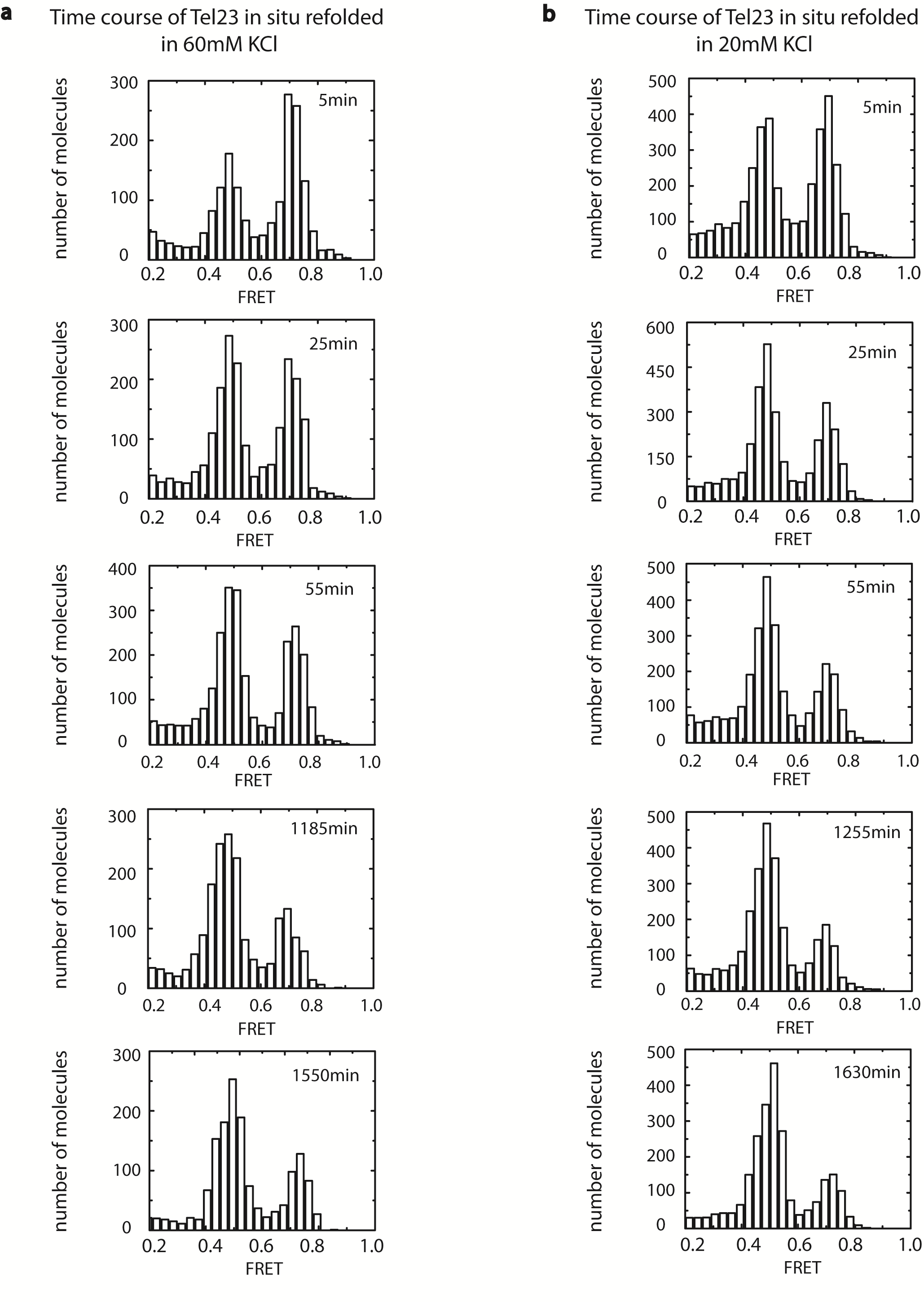


**Figure S3.** (**a**) Single-molecule FRET histograms of Tel23 *in situ* refolded in 60mM KCl for the indicated period of time. (**b**) Single-molecule FRET histograms of Tel23 *in situ* refolded in 20mM KCl for the indicated period of time.

**Figure S4**


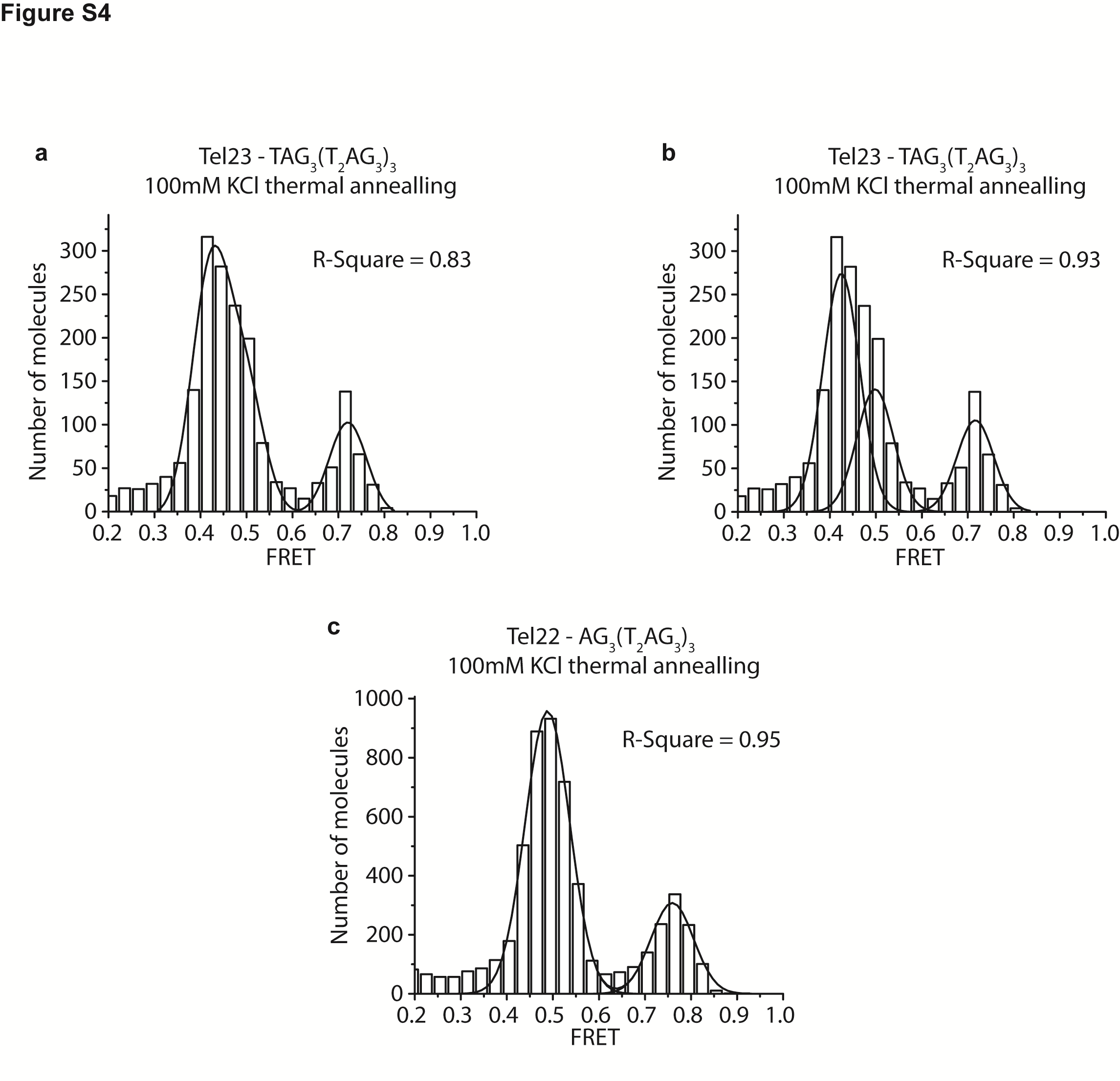


**Figure S4.** Gaussian fitting of smFRET distributions (a) Tel23 molecules thermally annealed and imaged in 100 mM KCl fit with two Gaussian functions centered at FRET=0.44 and FRET=0.72. (b) Tel23 molecules thermally annealed and imaged in 100 mM KCl fit with three Gaussian functions centered at FRET=0.42, FRET=0.50, and FRET=0.72. The addition of a third FRET population substantially improved the fitting results as indicated by the adjusted R^2^ value. (**c**) In contrast, measurements of Tel22 molecules, lacking the 5’ thymine, were well fit with only two FRET populations. This result highlights the effect of the 5’ thymine in promoting the folding of the hybrid structure as has been previously reported by NMR analysis[[2](#_ENREF_1)].

**Figure S5**


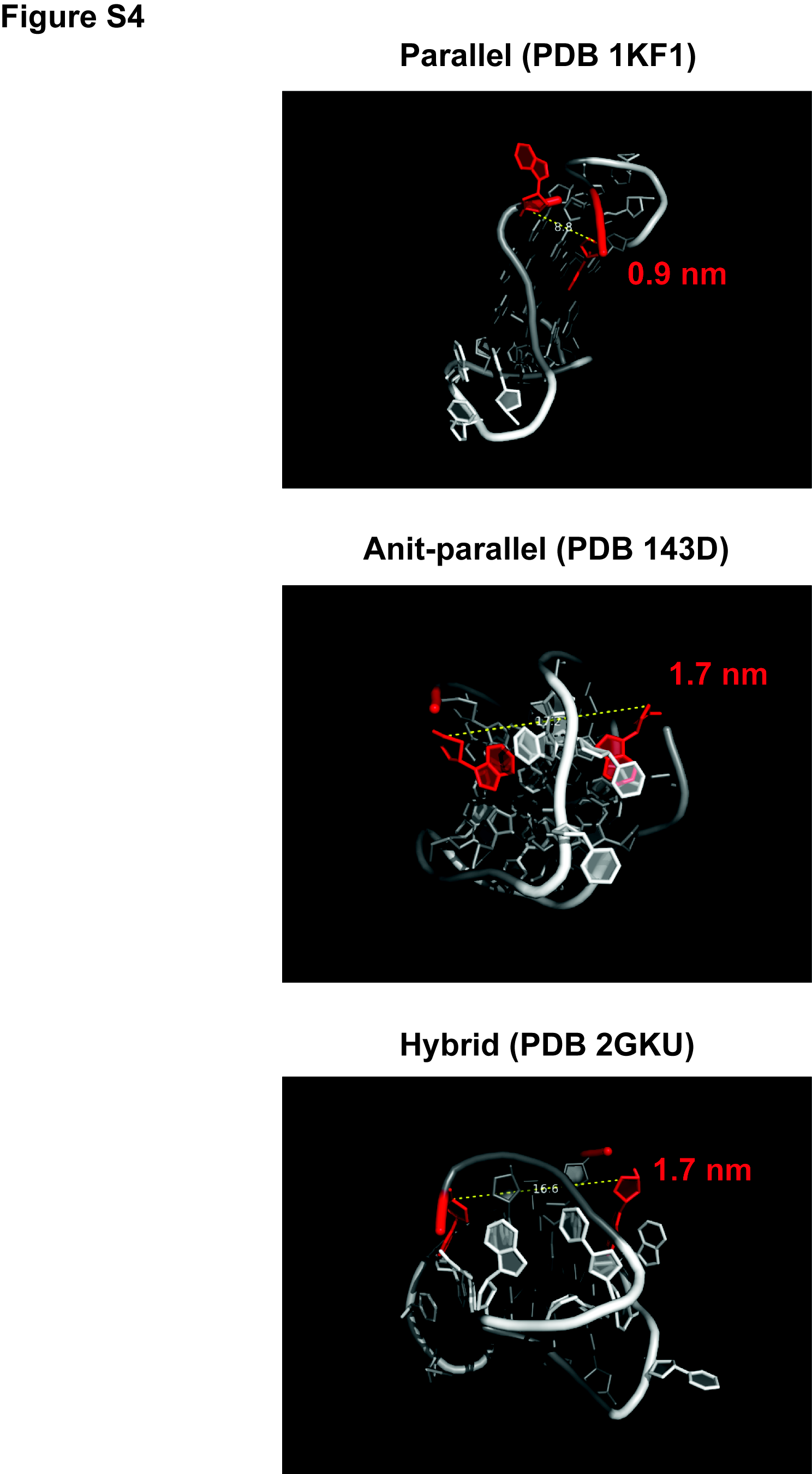


**Figure S5.** Atomic resolution structures of the parallel (top panel)[[3](#_ENREF_3)], anti-parallel (middle panel)[[4](#_ENREF_4)], and a hybrid form (bottom panel) [[2](#_ENREF_1)] telomere DNA GQ conformation. Measurements between the terminal deoxyribose moieties are indicated in red. Although a these measurements cannot be quantitatively compared with our smFRET results, they nevertheless are in good qualitative agreement with the assignements of the high-FRET state to the parallel conformation and the mid-FRET states to a mixture of the anti-parallel and hybrid conformations.

1. Jena PV, Shirude PS, Okumus B, Laxmi-Reddy K, Godde F, et al. (2009) G-quadruplex DNA bound by a synthetic ligand is highly dynamic. J Am Chem Soc 131: 12522-12523.

2. Luu KN, Phan AT, Kuryavyi V, Lacroix L, Patel DJ (2006) Structure of the human telomere in K+ solution: an intramolecular (3 + 1) G-quadruplex scaffold. J Am Chem Soc 128: 9963-9970.

3. Parkinson GN, Lee MPH, Neidle S (2002) Crystal structure of parallel quadruplexes from human telomeric DNA. Nature 417: 876-880.

4. Wang Y, Patel DJ (1993) Solution structure of the human telomeric repeat d[AG3(T2AG3)3] G-tetraplex. Structure 1: 263-282.
